# Supplementary material for: Billing rules / global period affect postoperative follow-up practices following total hip arthroplasty
Source: PLoS One. 2024 Apr 18;19(4):e0300460. doi: 10.1371/journal.pone.0300460 (PMC11025944; doi:10.1371/journal.pone.0300460)
Supplement: Attachment — Submitted filename: PLOS ONE Response to Reviewer.docx [file pone.0300460.s002.docx]

PLOS ONE Response to Reviewer/Editors

Thank you for taking the time to review our manuscript, “Billing rules / global period affect postoperative follow-up practices following total hip arthroplasty.” Please see responses to editor/reviewer queries below.

Response to Editor Comments:

1. Please ensure that your manuscript meets PLOS ONE's style requirements, including those for file naming.

**Response**: We have updated file names appropriately, thank you.

2. You indicated that ethical approval was not necessary for your study. We understand that the framework for ethical oversight requirements for studies of this type may differ depending on the setting and we would appreciate some further clarification regarding your research. Could you please provide further details on why your study is exempt from the need for approval and confirmation from your institutional review board or research ethics committee (e.g., in the form of a letter or email correspondence) that ethics review was not necessary for this study? Please include a copy of the correspondence as an ""Other"" file

**Response**: Our IRB listed the following statement as reason for the present project being exempt from IRB review “The Yale IRB determined that the investigator is not engaged in research involving human subjects. As such, IRB review and approval are not required.” We have attached a copy of the exemption form from our IRB to the submission as well. Thank you.

3. Note from Emily Chenette, Editor in Chief of PLOS ONE, and Iain Hrynaszkiewicz, Director of Open Research Solutions at PLOS: Did you know that depositing data in a repository is associated with up to a 25% citation advantage (https://doi.org/10.1371/journal.pone.0230416)? If you’ve not already done so, consider depositing your raw data in a repository to ensure your work is read, appreciated and cited by the largest possible audience. You’ll also earn an Accessible Data icon on your published paper if you deposit your data in any participating repository (https://plos.org/open-science/open-data/#accessible-data).

**Response**: Thank you for this information.

4. We note that your Data Availability Statement is currently as follows: All relevant data are within the manuscript and its supporting information files. Please confirm at this time whether or not your submission contains all raw data required to replicate the results of your study. Authors must share the “minimal data set” for their submission. PLOS defines the minimal data set to consist of the data required to replicate all study findings reported in the article, as well as related metadata and methods.

**Response**: We have attached an additional file containing raw data (supporting information file) used to build graphs for the present study. All data required to replicate study findings is present in the manuscript; however.

5. Please review your reference list to ensure that it is complete and correct. If you have cited papers that have been retracted, please include the rationale for doing so in the manuscript text, or remove these references and replace them with relevant current references. Any changes to the reference list should be mentioned in the rebuttal letter that accompanies your revised manuscript. If you need to cite a retracted article, indicate the article’s retracted status in the References list and also include a citation and full reference for the retraction notice.

**Response**: All references are accurate. None have been retracted.

Response to Reviewer Comments:

1. The study design does not allow for the establishment of causality and furthermore does not show this led to an increase in complications. If simple to do, looking at the same database and establishing if there were further complications requiring surgery for these patients by looking at procedure codes in the same timeline (or up to 2 years after) could potentially substantially add to the paper’s final discussion and conclusions and improve its clinical relevance.

**Response**: Thank you for this comment. Although the analysis you suggest could be interesting, our goal with the current manuscript is simply to characterize trends in the timing/provider type of follow-up visits and how this may relate to global billing practices. We do not aim to call into question the validity of current practices or suggest that current practices lead to inadequate patient care.

2. Administrative data was used which is vulnerable to coding inaccuracies. Only looking at visits coded with 99212-5 are not enough as there are multiple other reasons patients could be seen other than post-operative visits, such as planned contralateral hip arthroplasty or total knee arthroplasty. Can visit be associated with diagnoses of visit to limit this cofounder? There is also the possibility that the patients were seen by midlevel and surgeons in conjunction during the billing period but were less diligent about signing the notes.

Response: Thank you for these valid points. Unfortunately, we are unable to characterize diagnoses associated with follow-up visits due to database limitations. In efforts to reduce confounding, we have only included follow-up visits by relevant orthopedic surgeons. The proximity to index hip arthroplasty may also limit the likelihood that follow-up visits are for alternative reasons. The point you raise regarding both mid-levels and surgeons seeing patients during visits is also valid and is unfortunately another limitation of the present study. Despite this, the large variation in number of visits performed by non-surgeons and surgeons between the global and non-global periods highlights an overall trend in care and should negate individual billing errors. We have now included both factors you mention in the limitations section of the manuscript.

3. How missing data was managed could be expanded to better explain how these were addressed.

Response: We have further clarified our inclusion criteria in the methods of the study which only included patients whose surgeons billed under the global billing period and who follow-up data in the database for at least the 26-week postoperative period. Thus, all patients included in the final cohort had no missing data pertinent to the present study.

Possible grammatical/formatting errors noted
Line 42: “improve quality of life “–> “improve the quality of life“
Line 42: “individuals.(1, 2),(3)” –> “individuals.(1-3) “
Line 42: “for the similar services” –> “for similar services“
Line 143: “week 7 (5.2%) seems consistent” –> “week 7 (5.2%) seem consistent “

Response: We have made these corrections in the manuscript. Thank you.
